# The two missing pillars of ultra-processed food and health policy: food assistance and the first 1,000 days

**DOI:** 10.3389/fnut.2026.1912782

**Published:** 2026-10-20

**Authors:** Ting Liu, Zimeng Cheng, Bin Xue, Ankita Kumari, Abdul Rahaman

**Affiliations:** 1 School of Food & Pharmaceutical Science and Technology, Guangzhou College of Technology and Business, Guangzhou, China; 2 College of Food Science, South China Agricultural University, Guangzhou, Guangdong, China; 3 School of Food Science and Engineering, Foshan University, Foshan, China

**Keywords:** diet first 1000 days, health policy, human health, nutrition program, ultra-processed food

## Abstract

Global policies on ultra-processed foods (UPF) have largely focused on four major approaches: taxes and other fiscal measures, restrictions on food marketing, front-of-pack warning labels, and product reformulation. While these strategies are important, they mainly address foods sold through commercial markets and overlook two powerful public-sector influences on what people eat: food assistance programs and nutrition policies during the first 1,000 days of life. These systems shape the diets of more than one billion people worldwide, yet they remain largely absent from current UPF policy discussions. This gap matters because those most affected by food environments often have the fewest choices. Infants, young children, and families relying on public food programs do not always determine what foods are available to them; instead, their diets are shaped by program design, procurement decisions, and broader policy priorities. If UPF policies continue to focus only on consumer choice within retail markets, they risk leaving behind populations who are most dependent on institutional food systems. A more complete approach requires expanding UPF governance beyond commercial food environments, including extending the protective principles of the WHO Code of Marketing of Breast-milk Substitutes to the broader complementary feeding period up to 36 months and developing age-specific UPF definitions and thresholds tailored to infants and young children. This perspective argues that a comprehensive, life-course approach to food-system regulation and the reduction of preventable nutrition-related inequalities requires the explicit integration of these overlooked yet critical domains into existing UPF policy frameworks.

## 1 Introduction

Two of the most important food environments in people’s lives, public food assistance and nutrition during the first 1,000 days, still receive limited attention in the current UPF policy debate. This gap matters because these are settings in which food choices are often shaped less by individual preference than by cost, availability, program rules, caregiving demands, and developmental needs. If UPF policies fail to account for these realities, efforts to reduce the health burden associated with UPF consumption will remain incomplete and may continue to miss the people who have the least freedom to choose what they eat. Over the past decade, global UPF policy has begun to take a clearer shape. Inspired partly by lessons from tobacco and other public health regulatory approaches, governments and international organizations have focused on four main strategies: fiscal measures, restrictions on food marketing, front-of-pack warning labels, and product reformulation. Growing evidence, including the 2025 *Lancet* series on UPF and human health and recent policy analyses, has highlighted the potential of these approaches to reshape food environments and reduce harmful dietary exposures (1–4). Although the epidemiological evidence linking UPF consumption to adverse health outcomes is increasingly consistent, it is not methodologically uniform. A major umbrella review reported moderate-to-high heterogeneity in 21 of 45 pooled analyses, indicating substantial variation across populations, exposure definitions, study designs, and outcome assessment. Importantly, prediction intervals remained statistically significant for only a subset of outcomes, including all-cause and cardiovascular mortality, common mental disorders, Crohn’s disease, obesity, and type 2 diabetes, suggesting that some associations are more likely than others to persist across different settings. The review further identified excess-significance bias in approximately one-third of eligible pooled analyses and small-study effects in several associations, including those involving mortality, metabolic syndrome, obesity, and breast cancer (5, 6). Yet, the current UPF policy map has a clear boundary: it mainly focuses on foods that people purchase in commercial markets. Taxes, warning labels, marketing regulations, and reformulation policies primarily target supermarkets, restaurants, and online food platforms. These measures are important, but they assume that consumers are able to make choices within a food environment that responds to regulation. For many people, however, food choices are shaped long before they reach a shop or a digital marketplace (5–7).

Public food systems represent one such overlooked area. National food assistance programs, school meals, and institutional feeding systems influence what millions of people eat every day, particularly those who rely on these programs for regular access to food. Similarly, the first 1,000 days of life, from conception to a child’s second birthday, represent a critical period during which lifelong dietary patterns and health trajectories are shaped. Yet, policies governing this period have traditionally focused on breastfeeding, breast-milk substitutes, and complementary feeding, with limited consideration of the growing presence of UPFs in early-life diets (7, 8).

Both of these domains have changed dramatically as UPFs have become increasingly embedded in global food systems, but the policies guiding them have not evolved at the same pace. As a result, the very systems designed to support nutrition and health may unintentionally operate without considering UPF exposure. Addressing this gap requires expanding UPF policy beyond individual consumer choices and commercial markets to include the public institutions and early-life environments that fundamentally shape dietary patterns. Only by bringing these missing pillars into the UPF policy framework can we develop a truly comprehensive and equitable approach to protecting health across the life course (Figure 1).

**Figure 1:**
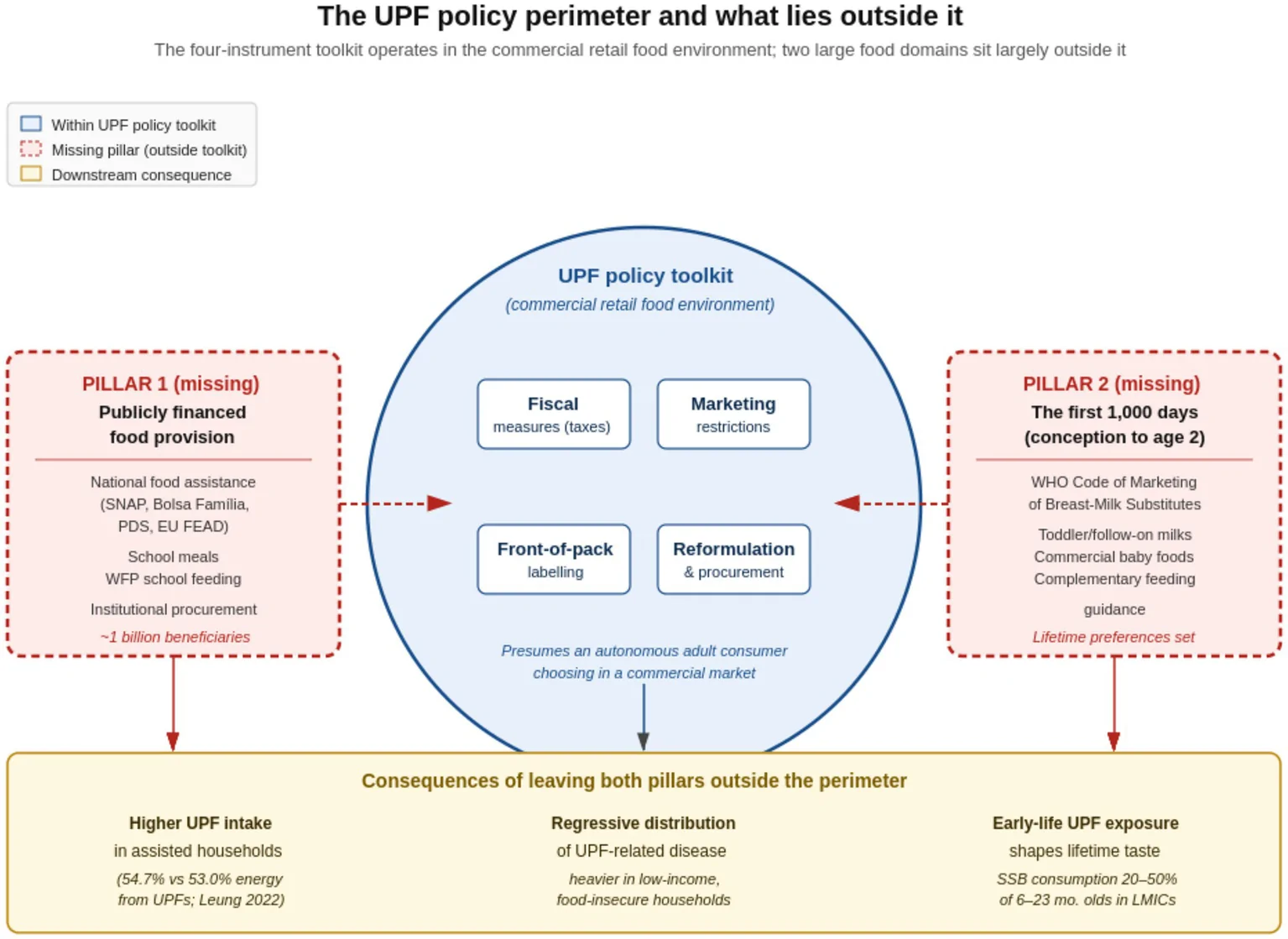
Conceptual framework illustrates that the current UPF policies largely target retail food environments, leaving publicly financed food provision and the first 1,000 days outside the policy perimeter.

## 2 Pillar one: food assistance as a structural channel facilitating UPF purchasing

National food assistance programs represent one of the most widespread forms of public nutrition support, reaching millions of households and shaping everyday food choices. In the United States, the Supplemental Nutrition Assistance Program (SNAP) is the largest program of its kind, supporting an average of 41.7 million individuals each month in fiscal year 2024, with federal expenditure reaching $99.8 billion (£78 billion; €92 billion) (9). However, SNAP was primarily established as an income-support mechanism to improve food access rather than as a structured intervention to promote dietary quality. Benefits are delivered through electronic benefit transfer (EBT) cards that can be used to purchase a broad range of foods and beverages from authorized retailers. Although certain categories, including alcohol, hot prepared foods, and dietary supplements, are excluded, many highly processed products, including sugar-sweetened beverages, packaged snacks, frozen meals such as pizzas, and refined breakfast cereals, remain eligible for purchase.

UPF consumption is a growing global concern, contributing approximately half or more of total dietary energy in many high-income countries while rising rapidly across low- and middle-income countries (LMICs). Evidence from Senegal, Kenya, India, Brazil, and Mediterranean populations links higher UPF intake with poorer diet quality, greater fat and free sugar intake, lower protein and micronutrient intake, and adverse metabolic outcomes. Of particular concern, UPF consumption is increasing among infants and young children in LMICs amid rising rates of childhood and maternal overweight, especially in poorer households. Early exposure to highly processed, sugary, and salty foods may shape long-term dietary preferences, reinforcing the need for stronger, context-specific policies on marketing, labeling, and the nutritional quality of foods intended for young children (10). Similar models are reflected in other large-scale food assistance systems, including Brazil’s Bolsa Família (11), Mexico’s former Prospera program (12), the World Food Programme’s school feeding initiatives, and the European Union’s Fund for European Aid to the Most Deprived (13, 14). Across these diverse settings, food assistance has largely focused on improving food availability and economic access. In contrast, considerations related to the level of food processing and exposure to ultra-processed foods have received comparatively limited policy attention.

By linking SNAP participation status with dietary recall data from two rounds of the “National Health and Nutrition Examination Survey,” Leung et al. identified a potentially pivotal finding. After accounting for socioeconomic and health-related characteristics, SNAP participants derived 54.7% of their total energy intake from UPF, compared with 53.0% for income-eligible non-participants; savory snacks and soft drinks were the primary drivers of this difference. Even more intriguing was the finding that SNAP participation modified the relationship between household food insecurity and UPF consumption (Figure 2A). Among non-participants, higher levels of food insecurity were associated with higher UPF consumption; however, this association was weaker among participants. This suggests that, across all levels of food security, SNAP participation does not fully protect individuals from the structural factors that drive UPF purchasing (15).

**Figure 2:**
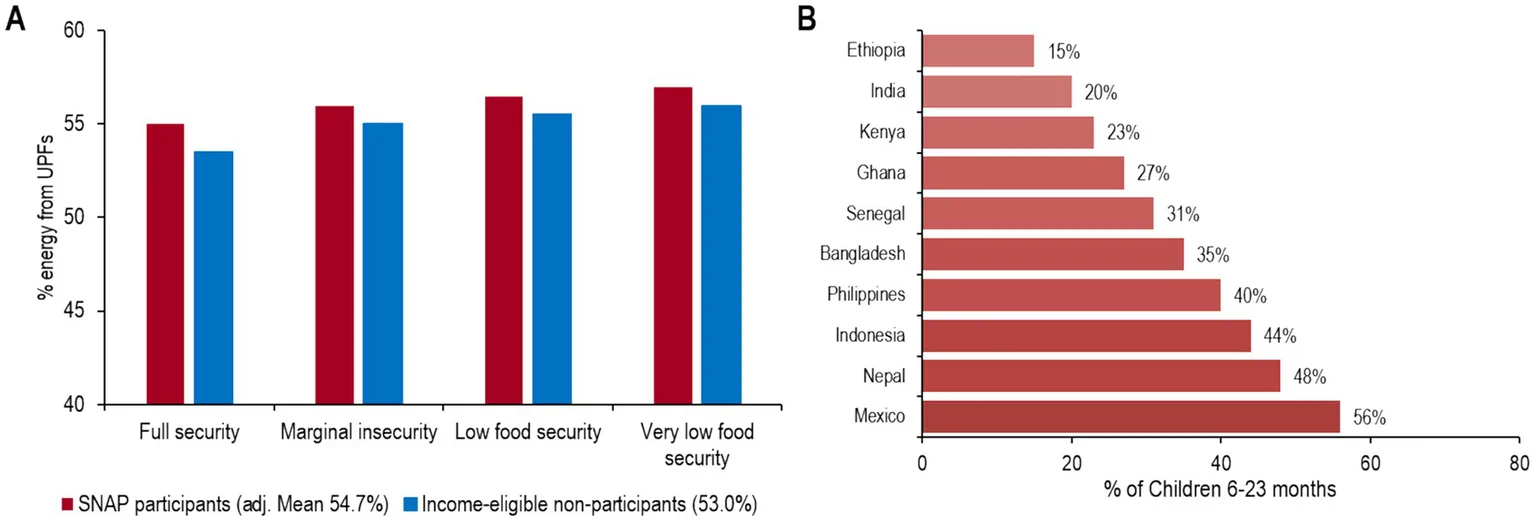
Parallel patterns across the two missing pillars. **(A)** Adjusted UPF intake by household food security and SNAP participation, independently prepared by the authors using selected data reported by Leung et al. (15) and publicly available NHANES data. **(B)** Sugar-sweetened beverage consumption among children aged 6–23 months in selected LMICs, adapted from Popkin and Laar (27) licensed under CC BY-NC 4.0.

This is not simply a question of individual dietary choice. Rather, it reflects how program design can shape the food environment in which choices are made. SNAP’s underlying architecture, including broad retailer eligibility, minimal product-level restrictions, and fixed monthly benefit cycles create a purchasing environment where inexpensive, shelf-stable, and energy-dense products can become the most convenient options, particularly when resources become limited toward the end of the benefit period (16, 17). In response to growing concerns about diet quality, 12 US states have received approval under Section 4001 of the Food and Nutrition Act to restrict the purchase of sugar-sweetened beverages, sweets, or both through SNAP, with implementation planned from 2026 onward (18). These policy changes represent an important opportunity for evaluation because they function as large-scale natural experiments. However, they also highlight a broader limitation of many UPF-focused policy approaches: restricting access to certain products does not necessarily improve the availability of healthier alternatives. When minimally processed foods are relatively expensive, geographically inaccessible, or difficult to prepare because of time constraints, limited cooking facilities, or household circumstances, as is often the case among food-insecure populations, removing selected products without simultaneously improving access to practical alternatives may unintentionally increase inequities. Effective reform therefore requires not only reducing exposure to less nutritious foods but also redesigning assistance programs to support affordable, accessible, and culturally appropriate healthier choices.

This limitation is illustrated by the contrast between restricting unhealthy purchases and actively enabling healthier choices. The Healthy Incentives Pilot, for example, provided SNAP participants with a 30-cent incentive for every SNAP dollar spent on targeted fruits and vegetables and demonstrated sustained increases in produce consumption (19). Microsimulation analyses further suggested that nationwide implementation of fruit and vegetable incentives within SNAP could prevent more than 300,000 cardiovascular events and reduce healthcare expenditures by approximately $6.8 billion (20). The implication is not that incentives are universally superior to restrictions, but that food assistance programs represent powerful policy instruments capable of shaping dietary patterns at the population scale. The critical question is therefore not whether these programs influence food choices, but the direction in which they are designed to operate. At present, the global default remains largely passive: assistance systems improve food access without adequately accounting for the health consequences of the foods that become most accessible. As a result, while UPF-focused taxation policies attempt to reduce consumption through price signals and generate revenue for public budgets, food assistance programs, often involving substantially larger public expenditures, may inadvertently channel significant purchasing power toward UPF-dominated food environments. Reorienting these programs toward healthier food systems represents a largely untapped opportunity within the global UPF policy landscape.

## 3 Pillar two: the first 1,000 days are outside the regulatory perimeter

The period from conception to 2 years of age is when lifelong dietary habits are established and when the impact of UPF on cognitive function and development is clearly evident (21, 22). This is also a period subject to extensive international nutrition policy and regulations, yet it is the least aligned with regulations specifically targeting UPFs. The “International Code of Marketing of Breast-milk Substitutes” was adopted by the World Health Assembly (WHA) in 1981 and has been further clarified through subsequent resolutions, such as WHA Resolution 78.18 (May 2025), regarding digital marketing (23). This Code prohibits the advertising of breast-milk substitutes, the distribution of free samples, direct contact between manufacturers and mothers, and the promotion of formula feeding as the norm. It stands as one of the most significant regulations limiting marketing in any sector. However, its current impact on UPF exposure is limited relative to the scale of the UPF problem. WHA Resolution 69.9 (2016) clarified that the Code applies to all milk products marketed for children up to 36 months of age, including follow-on and toddler milks. In fact, among the 11 East Asia-Pacific countries assessed in 2025, the Philippines and Thailand demonstrated the most comprehensive legal safeguards for this period (24). In the majority of countries, these regulations apply only up to 6 or 12 months of age. The fastest-growing segment of the commercial milk-formula market, toddler and “growing-up” milks for children aged 12 to 36 months, remains largely unregulated in most regions. These products often contain sugar at levels exceeding the recommended limits for added or free sugars in early childhood diets. The industry deliberately designs its packaging to resemble infant formula, creating a false impression of nutritional equivalence; yet these products are not subject to the same regulations (25).

Broadly speaking, commercial baby and toddler foods, such as pouches, snacks, cereals, and drinks for children aged 6 to 36 months, fall almost entirely outside the scope of measures targeting UPF. Front-of-pack warning labels, where they exist, were designed for the general public; taxes on sugary drinks often exempt or only partially cover beverages intended for infants and young children; and marketing restrictions aimed at children typically define “children” as those of primary school age or older. A recent analysis of commercial supplementary foods for children across seven Southeast Asian countries revealed that products marketed as beneficial for child development frequently involve ultra-processing and the use of additives (26). A recent analysis by Popkin and Ng, combining Demographic and Health Survey data with global sales figures, illustrates the serious and escalating impact of this trend (Figure 2B). In most of the low- and middle-income countries surveyed, the proportion of children aged 6 to 23 months who consumed a sugary drink on the day before the survey now ranges from one-fifth (20%) to more than half (over 50%) of the total. Global sales of sugar in foods for infants and young children rose from 1.75 trillion grams annually in 2010 to 2.41 trillion grams in 2023. During this period, the retail value of this sector rose from $35 billion to $74 billion (27). These are not the dietary habits of a population in which the “first 1,000 days” policy is being implemented as intended.

Two structural factors further exacerbate this disparity. First, the “first 1,000 days” agenda is institutionally distinct from the broader UPF policy discourse. It is embedded within the maternal and child health divisions of health ministries, the nutrition programs of UNICEF and WHO, and professional bodies representing obstetricians and pediatricians. Key analyses of UPF policy have failed to adequately incorporate these groups (2, 4, 28). Second, applying conventional UPF measures to products intended for infants is politically challenging: warning labels on baby food may provoke parental anxiety and trigger industry opposition, while taxes on beverages for young children can be perceived as “punishing parents.” Consequently, policy hesitation emerges precisely where the stakes for child development are highest.

## 4 Why the two pillars connect

These are not two separate or unrelated gaps; rather, they stem from a common structural feature. In both instances, the population in question does not operate in a vacuum but instead depends critically on external factors, such as the purchasing decisions made by others or the policy frameworks established by outside actors. Consider the SNAP participant: their food choices are not solely a matter of personal preference, but are significantly constrained and shaped by the eligibility rules governing EBT use, as well as by the realities of the local retail environment in which they shop. Similarly, the toddler’s dietary intake is mediated through the choices of their parent or caregiver, which are themselves influenced by pervasive industry marketing strategies, with those marketing practices in turn conditioned by the reach and limitations of the relevant regulatory code (29, 30). In both scenarios, the standard toolkit designed to address UPF consumption falls short because it implicitly assumes an autonomous adult consumer making independent decisions within a competitive commercial marketplace. As a result, this conventional approach captures only a fraction of the actual decision-making architecture, leaving the deeper structural influences largely unexamined and unaddressed.

A second connection is temporal because dietary preferences and metabolic trajectories begin to develop during the first 1,000 days of life and may influence later eating patterns and long-term disease risk (21, 31). This period is particularly relevant to food assistance policy, as families with children represent a substantial share of program participants; approximately 62% of SNAP participants live in households with children (9). Weaknesses in nutrition policies covering the first 1,000 days can therefore carry forward into households that later face the affordability, access, and food-environment constraints described under the first pillar, meaning that exposure to ultra-processed foods may occur after early taste preferences and dietary habits have already begun to take shape. The resulting socioeconomic gradient in UPF consumption, which is higher among lower-income and food-insecure populations and, in some settings, among households receiving food assistance, should therefore not be understood solely as a consequence of individual choice, because part of this pattern may reflect dietary exposures and structural conditions established during infancy and early childhood, long before children can make meaningful food choices for themselves.

A third connection is that the policy response in both domains has tended to be paternalistic rather than structural. Restricting what food-assistance recipients may buy, just as restricting what infant formula companies may say, treats consumption symptoms without altering the underlying availability and affordability of alternatives (32, 33). This is not an argument against either restriction. It is an argument that restriction without provision is, in food policy as elsewhere, a way of moving costs onto the people least able to absorb them.

## 5 A redesign agenda: integrating food assistance and early-life nutrition into the UPF policy framework

A meaningful response to the growing contribution of UPF to poor diet quality demands a broader policy approach, one that goes beyond consumer education, product reformulation, and piecemeal regulatory measures. Two critical policy areas remain underexplored in current UPF strategies: food assistance systems and nutrition protection during the first 1,000 days of life. These systems fundamentally shape the availability, affordability, and promotion of foods, especially among populations that are most exposed to unhealthy food environments. Moving forward, a comprehensive UPF policy framework should prioritize improving diet quality within food assistance programs, strengthening early-life nutrition safeguards, and building the evidence base necessary to inform effective, context-specific interventions.

### 5.1 Transform food assistance programs from improving food access to supporting healthy diets

Food assistance programs were historically designed to address hunger, poverty, and inadequate food access. However, in contemporary food environments, where inexpensive packaged foods are widely available, improving access to food does not necessarily guarantee access to healthy diets. The nutritional impact of these programs increasingly depends on whether their design supports or constrains healthier choices (34).

SNAP substantially improves food purchasing capacity among low-income households, but its primary function is economic support rather than dietary improvement. Most foods and beverages sold by authorized retailers remain eligible for purchase, with relatively limited consideration of the level of processing or overall dietary quality (3, 35). Similar patterns are observed in many food assistance systems globally, where programs have traditionally prioritized food security and affordability rather than exposure to ultra-processed foods (4).

However, improving food assistance does not necessarily require a restriction-only approach. Evidence from the US Healthy Incentives Pilot (HIP) demonstrates the potential of positive incentives. HIP provided SNAP participants with a financial incentive for purchasing fruits and vegetables and resulted in increased consumption of targeted produce among participants. Modeling studies have further suggested that expanding fruit and vegetable incentives nationally could produce substantial cardiovascular health benefits and healthcare savings (20, 36). These findings indicate that food assistance programs can serve as effective nutrition policy tools when they actively promote healthier food choices.

Nevertheless, incentives must be accompanied by broader structural changes. Research on food insecurity and SNAP participation suggests that reductions in diet quality toward the end of benefit periods are strongly associated with limited resources and insufficient benefit adequacy rather than simply with individual decision-making (16, 17). Therefore, effective redesign should combine adequate benefit levels, improved availability of affordable minimally processed foods, nutrition incentives, and retailer strategies that increase access to healthier options, particularly in underserved communities.

Rather than viewing food assistance and UPF reduction as separate policy areas, governments should recognize food assistance programs as potential platforms for improving population diet quality. The policy objective should not only be to reduce access to less healthy foods but also to ensure that healthier alternatives are economically realistic and practically achievable.

### 5.2 Extend UPF-related protection across the first 1,000 days of life

The first 1,000 days, spanning pregnancy through the second year of life, represent a critical period for establishing dietary patterns and influencing long-term health outcomes. While breastfeeding protection has received international attention through the WHO International Code of Marketing of Breast-milk Substitutes, regulatory attention to commercial complementary foods and toddler products remains comparatively limited (37). The rapid expansion of commercially marketed infant and toddler foods has created new challenges. Many products promoted for young children are highly processed and may contain added sugars, refined starches, or strong health-related marketing claims despite limited evidence that they provide advantages over minimally processed complementary foods (7, 38). This highlights a regulatory gap between existing infant feeding recommendations and the commercial food environment experienced by many families.

A stronger policy approach would extend protection beyond breastfeeding promotion to include the wider complementary feeding period. Priorities should include effective implementation of the WHO guidance on inappropriate promotion of foods for infants and young children, restrictions on misleading nutrition and health claims, and adoption of nutrient profiling systems to determine which products are suitable for promotion to young children (7, 37). Examples from broader food policy initiatives demonstrate that regulatory approaches can influence childhood food environments. Chile’s comprehensive food policy package, including mandatory front-of-pack warning labels and restrictions on child-directed marketing, provides one example of how governments can modify commercial food environments through coordinated action (39). Although such policies are not specifically limited to the first 1,000 days, they provide valuable lessons for protecting children from early exposure to unhealthy food marketing. Therefore, UPF policies should also ensure that early-life considerations are incorporated into broader measures, such as front-of-pack labelling, marketing restrictions, and fiscal policies. Young children should not be treated simply as smaller versions of adults; their nutritional requirements, developmental vulnerability, and exposure to commercial influence warrant specific consideration.

## 6 What would falsify this argument?

Despite growing attention to UPFs, important evidence gaps remain regarding how food assistance, early-life nutrition, and regulatory policies influence UPF exposure and health outcomes. Future research should rigorously evaluate emerging interventions, such as SNAP state-level waivers, assessing effects on purchasing, diet quality, food security, nutritional outcomes, and unintended consequences. Monitoring systems should also better capture UPF exposure among infants and young children, particularly from commercially produced foods, which remains insufficiently tracked. We have argued that food assistance and the first 1,000 days are missing pillars in UPF policy and that this absence has predictable, regressive consequences. The argument would be weakened by three kinds of evidence we currently lack. If a rigorously evaluated SNAP restriction policy produced sustained reductions in UPF intake without offsetting increases in food insecurity, it would suggest that restriction-only approaches may be more effective than we have assumed. If countries that legislatively extended code coverage to 36 months showed no measurable shift in toddler-targeted UPF consumption, it would suggest that the regulatory perimeter is less pivotal than we have argued. If the regressive distribution of UPF consumption proved more responsive to broad-spectrum instruments, such as taxes, labels, and marketing restrictions, than to food assistance redesign, that would shift the priority ordering. None of these results is currently in evidence.

The 2025 Lancet series and the parallel regulatory analyses have moved the UPF debate decisively past the question of whether the problem is real. The next move is to ask why the policy response, despite genuine progress, remains structurally incomplete. The answer, we have argued, runs through the public food sector and through the cradle. Until the toolkit reaches both, the policy architecture will keep delivering effects that are real but partial, and a distribution of harm that is predictable and unfair.

## 7 Conclusion

The global policy response to UPF has advanced considerably but remains structurally incomplete. By focusing almost exclusively on the commercial retail environment and the autonomous adult consumer, current UPF instruments, including fiscal measures, marketing restrictions, warning labels, and reformulation, have overlooked two domains that shape the diets of over a billion people: public food assistance programs and the first 1,000 days of life. Both function as structural amplifiers of UPF consumption rather than counterweights, with regressive consequences concentrated among populations whose food choices are most constrained. Redesigning food assistance around diet quality rather than caloric sufficiency, extending the WHO Code’s effective scope to 36 months, and embedding infant-and-toddler-specific thresholds into existing UPF tools are practical priorities. Without integrating these two missing pillars, UPF policy will remain partial, and the distribution of harm will remain predictable and unfair. Policy responses also require stronger coordination across health, social protection, agriculture, and food-regulation systems. Fragmented governance can weaken otherwise promising interventions, whereas integrated strategies can simultaneously address purchasing power, early dietary exposure, food availability, and marketing environments. Overall, reducing UPF-related inequalities requires shifting attention beyond individual choice toward the broader food systems and policies that shape what families can realistically access and consume.

## Data Availability

The original contributions presented in the study are included in the article/supplementary material, further inquiries can be directed to the corresponding authors.
